# Comparative Evaluation of Deep Learning Models for the Classification of Impacted Maxillary Canines on Panoramic Radiographs

**DOI:** 10.3390/diagnostics16020219

**Published:** 2026-01-09

**Authors:** Nazlı Tokatlı, Buket Erdem, Mustafa Özcan, Begüm Turan Maviş, Çağla Şar, Fulya Özdemir

**Affiliations:** 1 Department of Computer Engineering, Faculty of Engineering and Natural Sciences, Istanbul Health and Technology University, 34275 Istanbul, Turkey; 2 Department of Orthodontics, Faculty of Dentistry, Istanbul Health and Technology University, 34275 Istanbul, Turkey; buket.erdem@istun.edu.tr (B.E.); mustafa.ozcan@istun.edu.tr (M.Ö.); cagla.sar@istun.edu.tr (Ç.Ş.); fulya.ozdemir@istun.edu.tr (F.Ö.); 3 Department of Orthodontics, Faculty of Dentistry, Marmara University, 34722 Istanbul, Turkey; begumturanbt@gmail.com

**Keywords:** deep learning, radiography, panoramic, cuspid, tooth, impacted, artificial intelligence

## Abstract

**Background/Objectives:** The early and accurate identification of impacted teeth in the maxilla is critical for effective dental treatment planning. Traditional diagnostic methods relying on manual interpretation of radiographic images are often time-consuming and subject to variability. **Methods:** This study presents a deep learning-based approach for automated classification of impacted maxillary canines using panoramic radiographs. A comparative evaluation of four pre-trained convolutional neural network (CNN) architectures—ResNet50, Xception, InceptionV3, and VGG16—was conducted through transfer learning techniques. In this retrospective single-center study, the dataset comprised 694 annotated panoramic radiographs sourced from the archives of a university dental hospital, with a mildly imbalanced representation of impacted and non-impacted cases. Models were assessed using accuracy, precision, recall, specificity, and F1-score. **Results:** Among the tested architectures, VGG16 demonstrated superior performance, achieving an accuracy of 99.28% and an F1-score of 99.43%. Additionally, a prototype diagnostic interface was developed to demonstrate the potential for clinical application. **Conclusions:** The findings underscore the potential of deep learning models, particularly VGG16, in enhancing diagnostic workflows; however, further validation on diverse, multi-center datasets is required to confirm clinical generalizability.

## 1. Introduction

Impacted teeth are a common dental issue, especially in the upper jaw, with third molars and canines most often affected. Impacted maxillary canines occur in about 1% to 3% of people and can cause problems like malocclusion, root resorption of nearby teeth, cysts, and gum disease if not diagnosed or treated early [1,2]. Detecting these issues early and accurately is important for planning treatment. Diagnosis usually depends on manually reviewing two-dimensional X-rays or three-dimensional scans such as CBCT or MRI. These manual reviews take time and can vary between and within observers, which may affect how consistent the diagnosis is [3,4].

Artificial intelligence (AI), particularly convolutional neural networks (CNNs), has recently become a transformative tool in medical imaging [5]. While seminal works have established CNNs’ utility in dermatology and oncology [6,7], their application in dentistry has rapidly expanded to caries detection and periodontal assessment [8,9,10,11]. However, the specific domain of impacted tooth detection on panoramic radiographs presents unique challenges—such as overlapping dental structures and varying bone densities—that distinguish it from general feature extraction tasks.

Regarding impacted canines, the current literature is evolving but limited. Recent studies by Abdulkreem et al. [12] and Aljabri et al. [13] demonstrated the feasibility of using CNNs for impaction classification. Similarly, Minhas et al. [14] explored 3D reconstruction techniques. However, these studies often rely on single-architecture frameworks without a comparative benchmark of different feature extraction strategies. The lack of a direct comparison between varying CNN depths and designs (e.g., residual learning vs. depthwise separable convolutions) leaves a gap in understanding which architectural characteristics are optimal for this specific radiological task.

Consequently, the primary research question of this study is: Which pre-trained CNN architecture provides the optimal balance of sensitivity and specificity for detecting impacted maxillary canines on 2D panoramic radiographs? This study is grounded in the hypothesis that a deeper, yet architecturally simple network (such as VGG16) may offer superior feature extraction capabilities for dental textures compared to more complex, deeper networks that risk overfitting on moderate-sized medical datasets.

To test this hypothesis, we conducted a comprehensive comparative analysis of four distinct architectures—ResNet50, Xception, InceptionV3, and VGG16—adapted via transfer learning. Utilizing a clinically sourced dataset of 694 annotated images, this research aims to identify a robust solution that minimizes false negatives to support routine clinical practice.

The main contributions of this study can be summarized as follows:

- Comparative Analysis: We comprehensively evaluate four distinct CNN architectures (ResNet50, Xception, InceptionV3, and VGG16) for classifying impacted maxillary canines to identify the optimal feature extractor.
- High Performance Verification: We show that VGG16 achieves the highest diagnostic accuracy (99.28%) and a perfect recall rate, setting a new benchmark for this dental anomaly.
- Proof-of-Concept Visualization: Development of a diagnostic interface to visualize model predictions as a feasibility study, with clinical usability testing reserved for future research.
- Dataset Reliability: We use a clinically validated, mildly imbalanced dataset to ensure robust, unbiased model performance.

## 2. Materials and Methods

### 2.1. Study Design and Ethical Considerations

This retrospective study was conducted in accordance with the Declaration of Helsinki [15] and approved by the Institutional Review Board of Istanbul Health and Technology University (Approval No. 2024/08-03). All panoramic radiographs were anonymized prior to analysis to ensure patient confidentiality.

All images were obtained using a Planmeca ProMax 2D unit (Planmeca, Helsinki, Finland) with standardized exposure parameters of 66 kV and 5 mA. All images were obtained between June 2020 and June 2025.

Inclusion criteria required patients to be between 18 and 65 years of age and to have undergone panoramic radiography for routine dental assessment. Exclusion criteria comprised radiographs with significant artifacts such as metal scattering or patient movement, a history of previous maxillary surgeries, or incomplete imaging data. Data set population distribution information is shown in Table 1.

### 2.2. Dataset Acquisition

A total of 694 panoramic radiographs were retrospectively collected from the radiology department archives of Marmara University Faculty of Dentistry, spanning June 2020 and June 2025. The dataset included 396 scans with confirmed impacted maxillary canines and 298 scans without impactions.

To ensure robust ground truth labeling, all radiographs were independently evaluated by two experienced orthodontists, each with more than 10 years of clinical experience. The diagnosis of “impacted maxillary canine” was established using both radiographic findings and available clinical records. Disagreements between evaluators were resolved through consultation with a third senior specialist.

To prevent data leakage between training and validation sets, strict patient-level separation was implemented. Only one panoramic radiograph per patient was included in the study. As a result, random splitting of the dataset into training, validation, and test subsets ensured that no patient’s data appeared in more than one subset, thereby preserving the independence of the test set.

### 2.3. Image Preprocessing

All panoramic radiographs were resized to a uniform resolution of 224 × 224 pixels. This resolution was specifically selected to align with the standard input dimensions required by the pre-trained CNN architectures (ResNet50, Xception, InceptionV3, and VGG16), thereby preserving the spatial hierarchy of the pre-learned ImageNet weights during transfer learning. While downsampling reduces fine detail, this resolution offers an optimal trade-off between computational efficiency and the preservation of macroscopic diagnostic features necessary for detecting impaction patterns.

To address variability in radiographic exposure and contrast across multi-year archival data, a standardized preprocessing pipeline was implemented. The pipeline included grayscale normalization and contrast enhancement through histogram equalization to improve the visibility of bony structures and dental roots. Mild Gaussian filtering was then applied to reduce high-frequency noise while preserving essential edge definitions, such as cortical bone outlines and root contours.

To improve model generalization and mitigate overfitting, data augmentation techniques—including random rotation (±15°), horizontal flipping, and zooming (±10%)—were applied exclusively to the training dataset. The validation and test sets remained unaugmented to ensure unbiased performance evaluation. Furthermore, no manual cropping or region-of-interest (ROI) masking was performed; the full panoramic radiographs were utilized as input. This approach was chosen to evaluate the models’ capacity to identify affected canines within the global context of the dentomaxillofacial complex, simulating a fully automated screening workflow that does not require manual ROI selection.

### 2.4. Model Selection and Architecture

**Figure 1:**
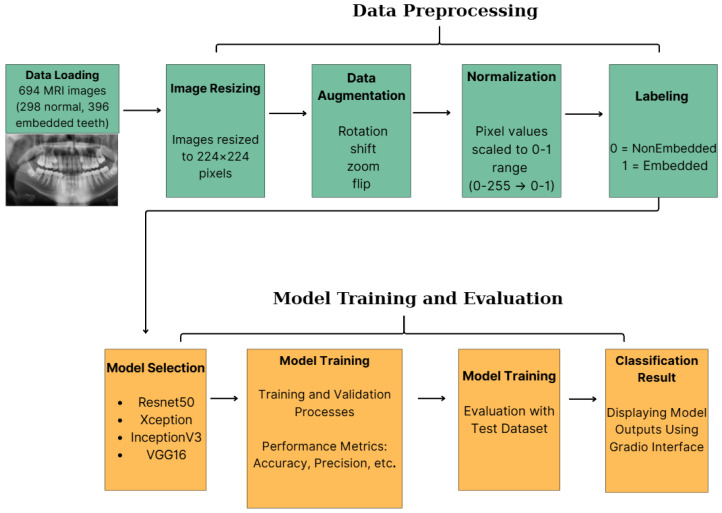
Data Preprocess, Model Training and Evaluation.

In this study, four state-of-the-art pre-trained convolutional neural network (CNN) architectures—ResNet50, Xception, InceptionV3, and VGG16—were selected for comparative analysis due to their established performance in medical image classification tasks [4,16]. The selection rationale was based on their distinct architectural characteristics: VGG16 was chosen for its deep yet simple stack of convolutional layers effective at capturing texture details; ResNet50 for its residual connections that mitigate vanishing gradients; InceptionV3 for its multi-scale feature extraction capabilities; and Xception for its parameter efficiency via depthwise separable convolutions. Figure 1 illustrates the general system architecture employed in this study.

### 2.5. Proposed Methodological Framework and Implementation Details

All models were implemented using the TensorFlow framework with the Keras API. A transfer learning strategy utilizing “feature extraction” was adopted. For all four architectures, the models were initialized with weights pre-trained on the ImageNet dataset, excluding the original top classification layers (‘include_top = False’). The convolutional base layers were explicitly set to be non-trainable (‘trainable = False’) to preserve the learned feature maps and prevent overfitting, given the dataset size.

A consistent custom classification head was appended to each frozen backbone, constructed sequentially as follows:

- A Global Average Pooling 2D layer to reduce the spatial dimensions of the feature maps.
- A Dense (Fully Connected) layer with 1024 units and ReLU activation to interpret the extracted features.
- A Dropout layer with a rate of 0.5 to enforce regularization.
- A final Dense layer with a single unit and Sigmoid activation to output the binary classification probability (p∈[0,1]).

To ensure reproducibility and isolate the impact of weight initialization, a fixed random seed of 42 was applied across all experimental runs. Training was executed in the Google Colab Pro environment, utilizing a high-performance NVIDIA A100 GPU to handle computational requirements.

All models were compiled using the Adam optimizer and binary cross-entropy loss. The training process was configured for a maximum of 15 epochs with a batch size of 32. To prevent overfitting and retain the optimal model parameters, an Early Stopping callback was employed, monitoring the validation loss with a patience of 3 epochs and configured to automatically restore the best model weights (‘restore_best_weights = True’) upon termination.

### 2.6. Proposed Methodological Framework and Transfer Learning Strategy

In contrast to traditional segmentation, which necessitates pixel-level annotations and considerable computational resources, this study introduces a direct classification framework optimized for efficient clinical screening. The proposed method comprises four steps: (1) region of interest (ROI) preprocessing, (2) feature extraction using pre-trained backbones, (3) dimensionality reduction, and (4) binary classification.

The selected architectures (ResNet50, Xception, InceptionV3, VGG16) were adapted for the specific task of detecting impacted canines using a tailored transfer learning strategy. The original top layers (fully connected layers) of each model, designed for 1000-class classification (ImageNet), were removed. The initial convolutional blocks were frozen to preserve low-level feature extractors (edges, textures), which are transferable across domains.

A custom classification head was then appended to each backbone, consisting of:

- A Global Average Pooling 2D layer to reduce spatial dimensions and minimize overfitting.
- A Dense layer with 512 units and ReLU activation to interpret high-level features.
- A Dropout layer (rate = 0.5) to further prevent overfitting during training.
- A Dense layer with a Sigmoid activation function to output a probability score p∈[0,1] indicating the presence of an impacted canine.

This approach uses a lightweight classification model instead of complex segmentation networks. It targets higher inference speeds for real-time integration with dental software.

To leverage prior knowledge from large-scale image datasets, transfer learning was employed by initializing each model with weights pre-trained on the ImageNet dataset, which contains over 1.2 million images across 1000 categories [17]. This approach enables efficient adaptation to domain-specific tasks, such as panoramic X-ray classification, even with relatively limited datasets. For each architecture, the final classification layers were modified to accommodate the binary classification task (presence or absence of impacted canines), wherein the original softmax activation was replaced with a sigmoid activation function to output probabilities suitable for binary outcomes.

- ResNet50 (Residual Network) introduces the concept of residual learning, which addresses the vanishing gradient problem commonly encountered in deep neural networks by incorporating identity shortcut connections. This design allows for the effective training of substantially deeper networks without degradation in performance [16]. ResNet50, consisting of 50 layers, has been widely adopted in medical imaging due to its capacity to capture complex hierarchical features.
- Xception (Extreme Inception) is an architecture based on depthwise separable convolutions, which factorize conventional convolutions into spatial and channel-wise operations. This results in a more efficient model with fewer parameters while maintaining high representational power [18]. Xception has demonstrated strong performance in tasks requiring fine-grained feature discrimination, making it suitable for nuanced medical image classification.
- InceptionV3 employs a sophisticated design involving parallel convolutional filters of varying sizes within its inception modules, enabling multi-scale feature extraction [19]. This architecture balances computational efficiency with depth, making it effective for complex image analysis tasks, including lesion detection and organ segmentation in clinical contexts.
- VGG16 is characterized by its simplicity and uniform architecture, utilizing sequential stacks of 3 × 3 convolutional layers followed by max-pooling layers [20,21]. Despite its relatively straightforward design, VGG16 has shown remarkable performance across various domains due to its deep structure (16 weight layers) and ability to capture detailed spatial hierarchies. Its robustness and ease of implementation make it a popular choice in medical imaging applications, particularly when working with smaller datasets.

By selecting these architectures, this study aims to provide a comprehensive evaluation of differing CNN design strategies in the context of detecting impacted maxillary canines. The fine-tuning process involved freezing the convolutional base layers to retain pre-trained feature representations, while the newly added dense layers were trained on the dental panoramic radiographs dataset to optimize classification performance for this specific diagnostic task.

#### Proposed Fine-Tuning and Custom Head Architecture

While the selected deep learning architectures (VGG16, Xception, ResNet50, and InceptionV3) are established in computer vision, our methodological novelty lies in the specific fine-tuning strategy and custom head architecture designed to optimize performance for the nuanced task of detecting impacted maxillary canines. To balance the extraction of high-level features learned from the ImageNet dataset with domain-specific adaptation, we implemented a targeted fine-tuning approach.

The convolutional base of each pre-trained model was frozen (trainable = False) to preserve the powerful, generalized feature extractors. This strategy is particularly effective in preventing overfitting when working with a specialized and limited medical imaging dataset (N=694) compared to the vast and diverse ImageNet dataset.

We then introduced a novel custom classification head, which was appended to each frozen backbone. This head consists of a Global Average Pooling 2D layer to reduce spatial dimensions and minimize overfitting, followed by a Dense layer with 1024 units and a ReLU activation function to interpret the extracted features, a Dropout layer with a rate of 0.5 for regularization, and a final Dense layer with a Sigmoid activation function.

This architecture was intentionally designed to create a lightweight, yet powerful, classifier that could be trained efficiently on top of the frozen layers, allowing for rapid adaptation of the pre-trained models to our specific diagnostic task. This combination of a frozen backbone with a custom-designed classification head represents a pragmatic and effective methodological enhancement for this clinical application.

### 2.7. Training and Validation Strategy

The dataset was partitioned into training (70%), validation (15%), and testing (15%) subsets using a single stratified split to preserve the class distribution (impacted vs. non-impacted) across all partitions. To ensure reproducibility and facilitate exact replication of the data splits, a fixed random seed of 42 was utilized.

The models were trained using the Adam optimizer with an initial learning rate of $1\times 10^{-4}$. The optimizer parameters were set to standard default values ($\beta_{1}=0.9$, $\beta_{2}=0.999$, and $\epsilon =1\times 10^{-7}$). The binary cross-entropy loss function was minimized during training.

Training was configured for a maximum of 15 epochs with a batch size of 32. To mitigate overfitting and optimize computational efficiency, an early stopping mechanism was implemented. This callback monitored the validation loss with a patience of 3 epochs, automatically terminating training if no improvement was observed. Crucially, the restore_best_weights parameter was set to True, ensuring that the model retained the weights corresponding to the lowest validation loss rather than the weights from the final epoch. The mild class imbalance (396 impacted vs. 298 non-impacted) was addressed primarily through the stratified sampling strategy during the dataset splitting phase, ensuring proportional representation in both training and evaluation sets without the application of synthetic oversampling or weighted loss functions.

### 2.8. Evaluation Metrics

Model performance was assessed using a comprehensive set of standard classification metrics to evaluate diagnostic effectiveness. Given the clinical significance of both false positives (potential overtreatment) and false negatives (missed diagnosis), specific emphasis was placed on metrics that reflect the balance between sensitivity and specificity.

For binary classification, the sigmoid output probabilities were converted to class labels using a standard decision threshold of 0.5. The computed metrics include Accuracy, Precision, Recall (Sensitivity), Specificity, and F1-Score [22,23]. Additionally, to evaluate the model’s discriminative ability across all possible classification thresholds—independent of the specific 0.5 cutoff—the Area Under the Receiver Operating Characteristic Curve (AUC-ROC) was utilized.

The metrics are defined as follows:

(1) $$Accuracy=\frac{TP+TN}{TP+TN+FP+FN}$$

Represents the overall proportion of correctly classified instances.

(2) $$Precision=\frac{TP}{TP+FP}$$

Indicates the reliability of positive predictions (Positive Predictive Value).

(3) $$Recall\left(Sensitivity\right)=\frac{TP}{TP+FN}$$

Measures the model’s ability to correctly identify all impacted canine cases.

(4) $$Specificity=\frac{TN}{TN+FP}$$

Reflects the ability to correctly identify non-impacted cases.

(5) $$F1\;Score=2\times \frac{Precision\times Recall}{Precision+Recall}$$

Provides the harmonic mean of Precision and Recall, offering a balanced view of performance in the presence of mild class imbalance.

(6) $$AUC\text{-}ROC=\int_{0}^{1}TPR\left(FPR^{-1}\left(t\right)\right)dt$$

Quantifies the probability that the model ranks a randomly chosen positive instance higher than a randomly chosen negative one, serving as a robust indicator of global diagnostic performance.

Where TP = True Positives, TN = True Negatives, FP = False Positives, FN = False Negatives, TPR = True Positive Rate, and FPR = False Positive Rate.

## 3. Results

### 3.1. Performance Metrics and Statistical Analysis

The classification performance of the four deep learning models—ResNet50, Xception, InceptionV3, and VGG16—was evaluated on the independent test set (N=104). To address the limitation of reporting only point estimates, 95% Confidence Intervals (CIs) were calculated for accuracy using the Wilson score interval method.

As summarized in Table 2, all architectures demonstrated robust diagnostic potential with accuracies exceeding 93%. However, VGG16 exhibited statistically superior performance compared to the other architectures. It achieved an accuracy of 99.28% (95% CI: 94.9–99.9), significantly outperforming Xception (93.53%, 95% CI: 87.0–96.8) and ResNet50 (94.24%, 95% CI: 88.1–97.3). Notably, VGG16 achieved a perfect Recall (Sensitivity) of 100%, indicating that it successfully identified all impacted canine cases in the test set without any false negatives.

### 3.2. Confusion Matrix and Error Analysis

To provide a granular analysis of misclassifications and rule out information leakage or overfitting, confusion matrices were generated for all models (Table 3). The error analysis reveals distinct patterns in how each architecture handles difficult cases.

The confusion matrix analysis underscores the clinical reliability of the VGG16 model. Specifically:

- False Negatives (Missed Diagnosis): VGG16 produced zero false negatives (FN = 0), meaning it did not miss a single case of an impacted canine. In contrast, Xception and InceptionV3 missed 2 cases each, and ResNet50 missed 1 case. In a clinical screening context, false negatives are the most critical error type, as they lead to untreated pathologies.
- False Positives (False Alarm): ResNet50 and Xception exhibited a higher tendency to classify non-impacted teeth as impacted, generating 5 false positives each. VGG16, conversely, generated only a single false positive (FP = 1), demonstrating superior specificity.

The disparity in error rates (1 error for VGG16 vs. 7 errors for Xception) supports the conclusion that VGG16’s simple yet deep architecture is better suited for extracting dental features from limited data compared to the more complex depthwise separable convolutions of Xception, which appeared to struggle with class separation in this specific dataset.

### 3.3. Training Dynamics and Convergence

**Figure 2:**
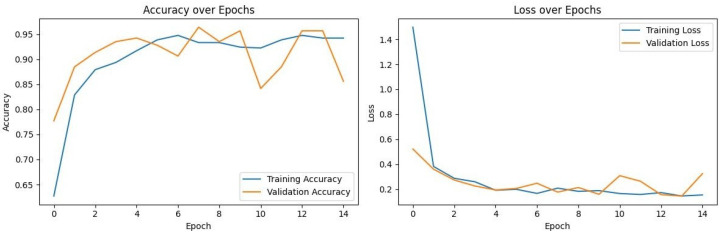
Training and Validation Performance of InceptionV3 over Epochs.

**Figure 3:**
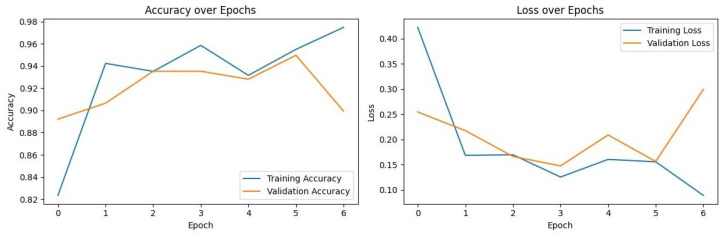
Training and Validation Performance of Xception over Epochs.

**Figure 4:**
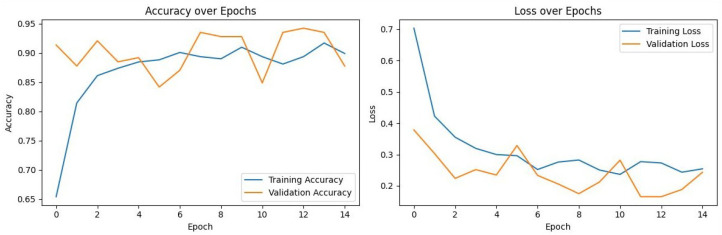
Training and Validation Performance of ResNet50 over Epochs.

**Figure 5:**
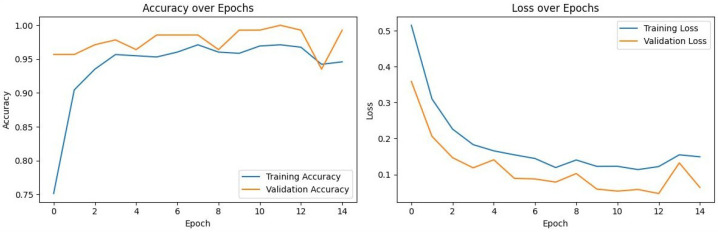
Training and Validation Performance of VGG16 over Epochs.

To assess model stability and verify that the high performance of the models was not a result of overfitting, the training and validation loss curves for each model were analyzed. Figure 2, Figure 3, Figure 4 and Figure 5 illustrate the performance of InceptionV3, Xception, ResNet50, and VGG16, respectively.

- VGG16 (Figure 5): Exhibited the most stable learning trajectory. Both training and validation accuracy increased consistently, while validation loss decreased steadily, indicating successful convergence without overfitting.
- Xception: Showed signs of early overfitting, as illustrated in Figure 3. The validation loss began to trend upward early in the process, which triggered the pre-configured Early Stopping mechanism at epoch 8. This is not an error, but rather an intended outcome of the training protocol, designed to prevent overfitting and retain the model at its point of peak generalization. The variation in training epochs across models is therefore a direct consequence of this automated regularization technique, with the Xception model converging on its optimal weights more rapidly than the other models before performance on the validation set began to degrade.
- ResNet50 (Figure 4) & InceptionV3 (Figure 2): Displayed fluctuations and spikes in the validation loss curves, suggesting instability in the optimization process compared to the smooth convergence of VGG16.

Collectively, these findings confirm that VGG16 achieved the optimal balance between bias and variance, delivering a highly generalizable model for the detection of impacted maxillary canines.

## 4. Discussion

The findings of this study demonstrate the efficacy of deep learning models, particularly convolutional neural networks (CNNs), in automating the detection of impacted maxillary canines from panoramic radiographs. Among the evaluated architectures, VGG16 achieved superior performance, attaining a perfect recall (100%) and an accuracy of 99.28%. While these metrics highlight the model’s potential utility in minimizing false negatives—a critical requirement for clinical screening tools—such high performance on a moderate-sized dataset warrants a critical interpretation regarding generalizability and potential overfitting to site-specific data characteristics.

### 4.1. Comparison with Prior Literature

The results indicate that the VGG16 model achieves superior performance (99.28% accuracy, 100% recall) compared to recent studies on canine impact detection. Relative to the findings of Alenezi et al. (2025) [24], a substantial performance gap is observed, particularly for the VGG16 architecture. In their study, VGG16 achieved only 83% accuracy and 78% sensitivity on a dataset of 182 panoramic radiographs. This difference is primarily attributable to dataset size. VGG16, as a deep architecture with approximately 138 million parameters, requires substantial data to generalize effectively. The use of a larger dataset (694 images) in the present study likely enabled the VGG16 model to overcome overfitting issues commonly observed in smaller datasets, thereby realizing its full potential for feature extraction in dental radiographs.

Additionally, Küçük et al. (2025) [4] reported high performance (96% F1-score) using a hybrid YOLO and Transformer-based approach for object detection. However, the classification-based approach in this study achieved a higher F1-score (99.43%). These results suggest that for the clinical task of screening patients for impacted canines, a robust classifier such as VGG16 may offer a more reliable and computationally efficient alternative to complex hybrid detection models.

A further methodological distinction involves preprocessing. Abdulkreem et al. (2024) [12] and Alenezi et al. (2025) [24] relied on automated cropping and landmark detection to enhance model performance, as their models (SqueezeNet, GoogLeNet) exhibited limitations with full panoramic images. In contrast, the present study demonstrates that VGG16, when trained on a sufficiently large dataset and utilizing standard image enhancement techniques such as histogram equalization, can effectively identify impacted teeth from full panoramic views without complex pre-cropping steps. This approach streamlines the clinical workflow and reduces the computational overhead associated with multi-stage detection pipelines.

The architectural framework and the quantitative performance of our proposed VGG16 model are compared with deep learning studies on impacted tooth detection in Table 4 and Table 5, respectively.

### 4.2. Architectural Performance and Training Dynamics

The superior performance of VGG16 over ResNet50 and Xception is supported by the training dynamics observed in the Section 3. While transfer learning provided a strong initialization for all models, the validation loss curves indicated that Xception and ResNet50 exhibited signs of optimization instability and early overfitting, likely due to their high parameter complexity relative to the dataset size (N=699). In contrast, VGG16 demonstrated smooth convergence, suggesting that its architecture offers a more favorable inductive bias for the specific feature sets of panoramic radiographs (e.g., tooth angulation and root resorption patterns) within a limited data regime.

### 4.3. Limitations and Sources of Bias

Despite the promising results, several critical limitations inherent to the retrospective, single-center design must be addressed. First, the dataset size (N=699), while sufficient for transfer learning, is relatively small compared to large-scale non-medical datasets. The reporting of 100% recall in the test set, while encouraging, raises the possibility of “spectrum bias,” where the study population (patients at a university hospital) may present with more distinct or severe impactions than a general dental practice population.

Secondly, as the data were sourced from a single institution using a single radiographic device (Planmeca ProMax 2D), the models may have learned device-specific artifacts or noise distributions (“shortcut learning”) rather than purely anatomical features. Consequently, the performance metrics reported here should be viewed as an internal validation; significant drops in accuracy could occur if the model is applied to images from different manufacturers (e.g., Sirona, Carestream) without retraining.

Thirdly, the text in earlier drafts mentioned comparing “two approaches,” which was an inconsistency; this study explicitly compared four architectures to ensure a robust evaluation. Finally, demographic skew is a potential concern; although the dataset was balanced for gender, age-related bone density variations could influence model predictions, a factor not explicitly controlled for in this analysis.

### 4.4. Clinical Integration and Future Directions

To explore the translational potential of this technology, a Gradio-based interface was developed as a proof-of-concept. It is important to clarify that this interface is currently a visualization prototype and has not undergone prospective clinical testing. Real-world deployment would require integration into Picture Archiving and Communication Systems (PACS) and rigorous evaluation of human-computer interaction to ensure it aids, rather than distracts, clinicians.

Future work must focus on external validation using multi-center datasets to overcome the described single-center biases. Additionally, while extending this work to 3D modalities like CBCT is a logical next step, it presents a significant “domain shift” challenge. The volumetric nature of CBCT requires fundamentally different 3D-CNN architectures (e.g., 3D U-Net) and far greater computational resources than the 2D classification framework presented here.

## 5. Conclusions

The present study demonstrates the feasibility of applying deep learning algorithms for the automated detection of impacted maxillary canines on panoramic radiographs within a controlled, single-center dataset. Among the evaluated architectures, VGG16 achieved an accuracy of 99.28% and a recall of 100% during internal testing.

**Figure A1:**
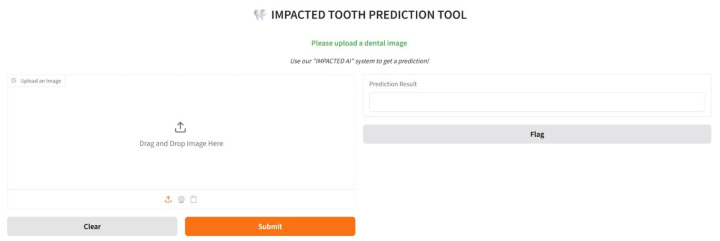
Application Main Screen.

As an initial step toward clinical translation, the top-performing model was integrated into a proof-of-concept diagnostic interface using the Gradio library (version 4.44.0) [26] as shown in Appendix A (Figure A1). This interface currently serves to visualize model predictions and has not yet undergone clinical usability testing.

Although these findings indicate that deep learning models may assist in diagnostic workflows, the results should be interpreted in light of the study’s retrospective, single-center design. To establish clinical generalizability and robustness, future research should prioritize external validation using diverse, multi-center datasets. Additionally, expanding the methodological framework to incorporate three-dimensional imaging modalities, such as CBCT, and conducting prospective clinical trials are essential prerequisites for broad clinical adoption.

## Figures and Tables

**Table 1:** Demographic Characteristics of the Study Population.

Sex	*n* (%)	Min Age	Max Age	Mean ± SD
Female	384 (55.3%)	18.33	65.58	24.48 ± 10.35
Male	310 (44.7%)	18.67	64.58	26.08 ± 11.27

**Table 2:** Performance comparison of CNN models on the test set (*N* = 104) with 95% Confidence Intervals.

Model	Accuracy (%)	95% CI	Precision (%)	Recall (%)	F1-Score (%)
ResNet50	94.24	90.4–98.1	92.55	98.86	95.60
Xception	93.53	89.5–97.5	92.47	97.73	95.03
InceptionV3	95.68	92.3–99.0	95.56	97.73	96.63
VGG16	99.28	94.9–99.9	98.88	100.00	99.43

**Table 3:** Confusion Matrix values for each model (*N* = 104). TP: True Positive, TN: True Negative, FP: False Positive, FN: False Negative.

Model	TN	FP	FN	TP	Total Errors
ResNet50	33	5	1	65	6
Xception	33	5	2	64	7
InceptionV3	35	3	2	64	5
VGG16	37	1	0	66	1

**Table 4:** Comparison of the proposed methodology with recent deep learning studies on impacted tooth detection (2022–2025).

Study	Target Task	Dataset Size	Core Architecture
Alenezi et al. (2025) [24]	Classification	182	GoogLeNet
Küçük et al. (2025) [4]	Object Detection	407	YOLOv8 + RT-DETR
Veerabhadrappa (2025) [25]	Detection (3rd Molar)	1100	VGG16 + YOLOv7
Abdulkreem et al. (2024) [12]	Classification	182	SqueezeNet
Aljabri et al. (2022) [13]	Classification	416	Inception V3
Current Study	Classification	694	VGG16

**Table 5:** Quantitative performance comparison of the proposed VGG16 model with state-of-the-art methods reported in the literature.

Study	Best Model	Accuracy	Recall	F1-Score
Küçük et al. (2025) [4]	Hybrid YOLO	–	99.20%	96.00%
Alenezi et al. (2025) [24]	GoogLeNet	94.00%	91.00%	–
Alenezi et al. (2025) [24] *	VGG16	83.00%	78.00%	–
Veerabhadrappa (2025) [25]	VGG16	93.51%	89.47%	91.97%
Aljabri et al. (2022) [13]	Inception V3	92.59%	–	–
Current Study	VGG16	99.28%	100.00%	99.43%

* Represents the performance of VGG16 on a smaller dataset (*N* = 182) as reported by Alenezi et al. [24].

## Data Availability

The data presented in this study were obtained from the archives of Marmara University Faculty of Dentistry. The data are not publicly available due to patient privacy restrictions but may be available from the authors upon reasonable request.

## Abbreviations

The following abbreviations are used in this manuscript:

AI	Artificial Intelligence
CBCT	Cone-Beam Computed Tomography
CNN	Convolutional Neural Network
DL	Deep Learning
FN	False Negatives
FP	False Positives
FPR	False Positive Rate
MRI	Magnetic Resonance Imaging
TN	True Negatives
TP	True Positives

## Appendix A

This interface was developed using the Gradio library (version 4.44.0) to showcase the prediction capabilities of the trained deep learning models in a user-friendly environment. Its primary purpose was to allow users to upload a dental X-ray image and instantly view the model’s prediction on whether an impacted canine is present, along with its confidence score. The interface was created as a crucial component that transforms the project’s theoretical aspect (model training) into a practical application, effectively visualizing the model’s performance on real-world data.
